# DAE-ConvBiLSTM: End-to-end learning single-lead electrocardiogram signal for heart abnormalities detection

**DOI:** 10.1371/journal.pone.0277932

**Published:** 2022-12-30

**Authors:** Bambang Tutuko, Annisa Darmawahyuni, Siti Nurmaini, Alexander Edo Tondas, Muhammad Naufal Rachmatullah, Samuel Benedict Putra Teguh, Firdaus Firdaus, Ade Iriani Sapitri, Rossi Passarella

**Affiliations:** 1 Intelligent System Research Group, Faculty of Computer Science, Universitas Sriwijaya, Palembang, Indonesia; 2 Department of Cardiology & Vascular Medicine, Dr. Mohammad Hoesin Hospital, Palembang, Indonesia; Menoufia University, EGYPT

## Abstract

**Background:**

The electrocardiogram (ECG) is a widely used diagnostic that observes the heart activities of patients to ascertain a heart abnormality diagnosis. The artifacts or noises are primarily associated with the problem of ECG signal processing. Conventional denoising techniques have been proposed in previous literature; however, some lacks, such as the determination of suitable wavelet basis function and threshold, can be a time-consuming process. This paper presents end-to-end learning using a denoising auto-encoder (DAE) for denoising algorithms and convolutional-bidirectional long short-term memory (ConvBiLSTM) for ECG delineation to classify ECG waveforms in terms of the PQRST-wave and isoelectric lines. The denoising reconstruction using unsupervised learning based on the encoder-decoder process can be proposed to improve the drawbacks. First, The ECG signals are reduced to a low-dimensional vector in the encoder. Second, the decoder reconstructed the signals. The last, the reconstructed signals of ECG can be processed to ConvBiLSTM. The proposed architecture of DAE-ConvBiLSTM is the end-to-end diagnosis of heart abnormality detection.

**Results:**

As a result, the performance of DAE-ConvBiLSTM has obtained an average of above 98.59% accuracy, sensitivity, specificity, precision, and *F1* score from the existing studies. The DAE-ConvBiLSTM has also experimented with detecting T-wave (due to ventricular repolarisation) morphology abnormalities.

**Conclusion:**

The development architecture for detecting heart abnormalities using an unsupervised learning DAE and supervised learning ConvBiLSTM can be proposed for an end-to-end learning algorithm. In the future, the precise accuracy of the ECG main waveform will affect heart abnormalities detection in clinical practice.

## Introduction

Heart abnormality (HA) was responsible for less than one-tenth of all deaths worldwide at the beginning of the 20^th^ century [1]. Global HA deaths are projected to increase to 23.4 million, comprising 35% of all deaths in 2030 [2]. Medical practitioners analyse information about the electrical function of the heart via electrocardiogram (ECG) signals. The electrocardiogram is a non-invasive, economic primary tool that can be used to diagnose HA [3–5]. The signals come from the electrodes placed on the patient’s limbs and the surface of the chest [3]. Relevant information from the ECG must be extracted from the physiological signal to support a specific healthcare application [6]. However, noise or artifacts are merged with the ECG signal, making it hard for the physicians to ascertain a true diagnosis. The unwanted signals encountered in ECG signals include powerline interference, baseline wander, electrode motion artifacts, and electromyographic noise [7]. These ontologies are unavoidable to contain conflicts and inconsistencies in physicians’ observations. The changes in ECG waveforms indicate an illness of the cardiac system that may occur for any reason. ECG signals are enhanced by removing various noises and artifacts to avoid misunderstanding. As a result, applying adequate signal processing methods is beneficial in eliminating noise from ECG signals.

Several signal processing applications, such as denoising, have been implemented in many works of literature [8–11]. ECG signal denoising aims to eliminate as much noise as feasible while preserving as much signal as possible. Daqrouq used discrete wavelet transform (DWT) to reduce the ECG baseline wandering [12]. Discrete wavelet transform is used for ECG signal pre-processing because of the properties of good representation, nonstationary signals and the possibility of dividing the ECG signal into different frequency bands. Sayadi and Shamsollahi [8] presented the adaptive bionic wavelet transform (BWT) for ECG baseline correction. The resolution in the time-frequency domain can be adaptively adjusted, not only by the signal frequency but also by the signal instantaneous amplitude and its first-order differential. Jenkal *et al*. [9] explored DWT to improve the filtering of the ECG signal. The study combines the DWT and an efficient method using the adaptive dual threshold filter (ADTF). In the results, the ADTF-DWT method offered high performance compared to an adaptive algorithm using a mean filter [10] and an ADTF and Riemann–Liouville integral [13]. Fasano *et al*. [14] intended to preserve the ST-segment when removing baseline wander. The study carries out an approach based on quadratic variation reduction. The quadratic variation is a well-known property used to analyse stochastic processes. Kaur *et al*. [15] used parameters such as power spectral density (PSD), average power, and signal-to-noise ratio (SNR), which calculated signals to compare the performance of different filtering methods. In addition, some studies propose the implementation of wavelet networks for ECG noise reduction. Zhang and Benveniste [16] first introduced the performance of wavelet networks. Poungponsri and Yu [17] presented an adaptive filtering technique based on wavelet transform and an artificial neural network for ECG signal noise reduction. The neural network employed in this approach performs the inverse wavelet transform (IWT) for signal reconstruction and also serves as a nonlinear adaptive filter to further reduce noise.

The applications mentioned above for signal denoising are capable of reducing ECG noise. Outside of the ECG signal frequency band, an adaptive filter can properly eliminate noise; nevertheless, it will fail when the signal and noise have the same frequency range. Also, by shrinking the wavelet coefficients in the transformed domain, the wavelet transform can effectively suppress noise. Unfortunately, obtaining a suitable wavelet basis function and threshold technique requires prior information, which is time-consuming in practice [18]. Therefore, those analyses show that there are still chances to improve the conventional ECG denoising techniques further.

Recently, denoising algorithms based on deep learning (DL) have been explored for performing the ECG signal denoising [19–23]. The deep learning algorithms can generalise for the scenarios of numerous noises using a single model. Those models learn their parameters for different noisy conditions, and an individual model can denoise various noises [24]. As computing power improves, many DL algorithm-based ECG denoising studies appear promising due to their improved generalisation capacity in various noise scenarios. The signal denoising algorithm based on the denoising auto-encoder (DAE) has had an outstanding performance compared to conventional denoising algorithms [19–23]. The DAE, a variant of the auto-encoder (AE), is composed of encoding and decoding layers; the encoding layer keeps the lower dimensional representation in the hidden layer, and the decoding layer extracts features to reconstruct the input [25]. With the excellent performance of the DAE to enhance the ECG signal conditions from noise and artifacts, this study aims to combine the DAE as the ECG denoising technique with our previous model, ConvBiLSTM, for detecting heart abnormalities [26]. The convolution layer as the feature extraction, part of convolutional neural networks (CNN) [27, 28], focused solely on one-dimensional ECG signal data. BiLSTM can be proposed as the classifier with both forward and backward phases to predict the ECG waveform (P-wave, QRS-complex, T-wave and isoelectric lines).

The paper’s structure follows: Section 2 discusses the experimented data, DAE-ConvBiLSTM architecture, and used hyperparameters. Section 3 presents the results and visualisation of the reconstructed ECG signal using trained DAE and the performance of ECG delineation based on ConvBiLSTM. In the last section, we offer the conclusion.

### The contributions of the study

In our previous study [26], we generated the stacking of the convolutional layer and the bidirectional long short-term memory (BiLSTM) for delineating the ECG waveform. In the instant study, we combined the DAE-ConvBiLSTM to detect heart abnormalities. We propose a novel denoising algorithm for ECG signals utilising ConvBiLSTM. The study’s contributions can be summarised as follows:

- To propose end-to-end learning using DAE for single-lead ECG signal denoising and ConvBiLSTM for delineating the ECG waveform. DAE will be optimised to eliminate noise from ECG signals because it was trained on ECG data. ConvBiLSTM, a hybrid deep learning model, has been proposed to classify the waveform of P-wave, QRS-complex, T-wave, and isoelectric lines.
- To detect heart abnormalities in the ECG surface by T-wave (due to ventricular repolarisation) observation to identify abnormalities and diseases associated with it.

## Material and method

**Fig 1:**
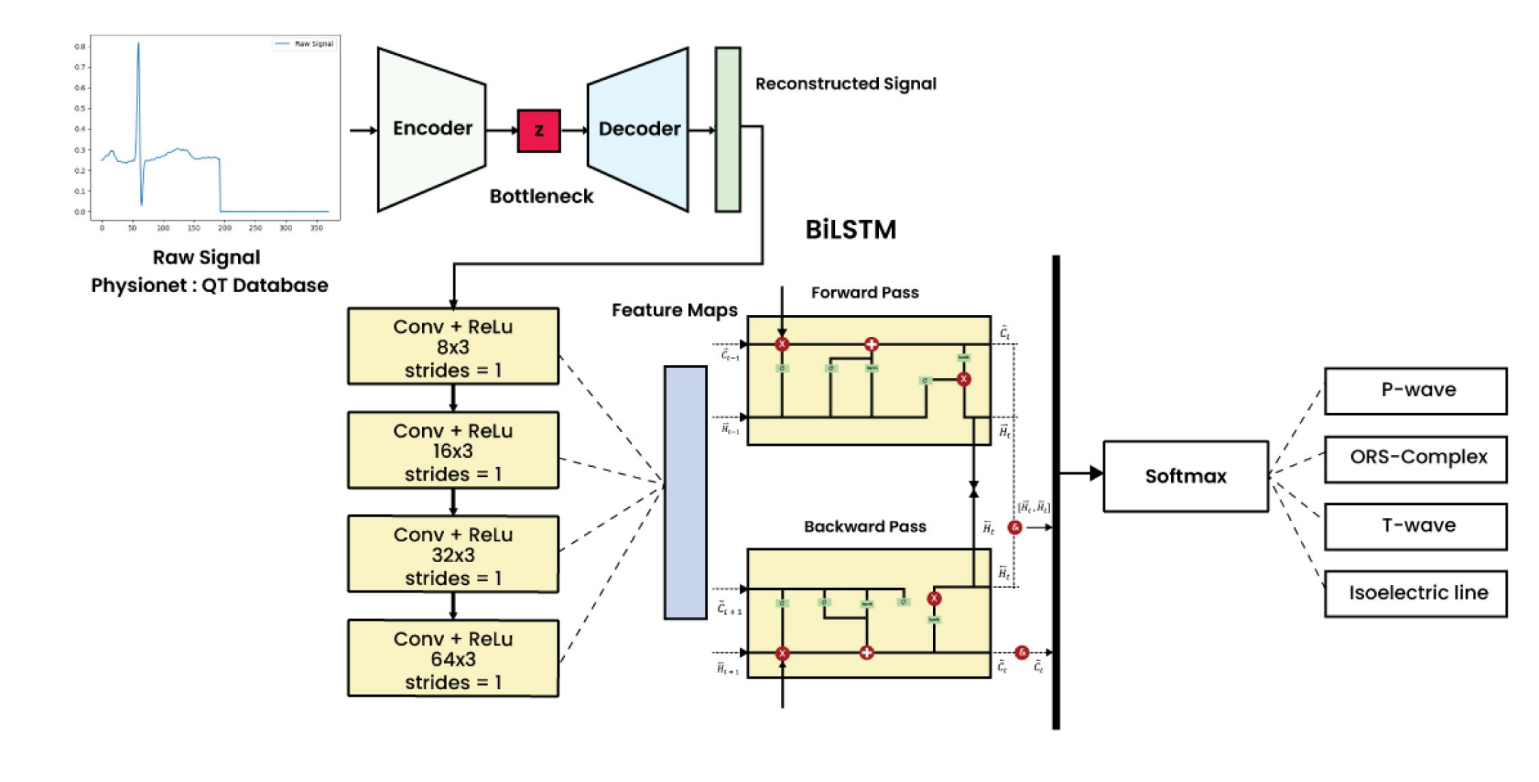
The proposed methodology of DAE-ConvBiLSTM.

The proposed methodology of the manuscript can be presented in Fig 1. The raw ECG signal of QTDB has been experimented with for a delineation task. The raw data has been denoised using the encoder and decoder phases using DAE. The reconstructed signals are the input for ECG feature extraction in convolution layers. The representation of feature maps can be calculated in forward and backward stages to classify the P-wave, QRS-complex, T-wave and Isoelectric line.

### Data preparation

The QT Database (QTDB) has been widely explored to generate the ECG delineation model, i.e., DAE-ConvBiLSTM [29]. The QTDB has 105 records, all digitized at 250 Hz. Among the records, the MIT-BIH Normal Sinus Rhythm Database were the only one experimented on in this study due to its complete and normal ECG waveform pattern. The QTDB contained a beginning (onset), peak, and end (offset) of each of the P-wave, QRS-complex, and T-wave in signals 0 and 1, respectively, using the *ecgpuwave*.

After the ECG delineating process using the DAE-ConvBiLSTM model, the model was used to detect the presence of T-wave alternans (TWA). The T-Wave Alternans Challenge Database is a challenge database for TWA identification with a wide variety of data that might be appropriate for the challenge [30]. There are 100 records sampled at 500 Hz. All records include patients with heart abnormalities and other risk factors and synthetic cases with calibrated amounts of TWA. The summary of data preparation can be seen in Table 1.

**Table 1:** ECG database description.

Dataset	Frequency sampling	Number of records	Description
Physionet: QT Database (MIT-BIH Normal Sinus Rhythm Database)	250 Hz	10	Delineation model for training and validation test
T-Wave Alternans Challenge Database	500 Hz	100	TWA Quantification

### DAE-ConvBiLSTM

The AE comprises one input, one hidden, and one output layer. The encoder and decoder are the basic architecture of an AE. The AE takes unlabeled inputs, encodes these inputs, and subsequently reconstructs the inputs as precisely as possible [6]. Vincent *et al*. [31] originally invented a variant of the classic AE applied in ECG processing tasks, denoising auto-encoder (DAE). The DAE was first explored to obtain robust features from a corrupted input and play the role of denoising. The DAE created a corrupted copy of the input by introducing some noise. Denoising refers to intentionally adding noise to the raw input before providing it to the network. Unlike AE, DAE had to remove the corruption to generate an output similar to the input. The initial input, *x*, is corrupted to $\tilde{x}$ by a stochastic mapping $\tilde{x}∼q(\tilde{x}|x)$. The DAE used corrupted $\tilde{x}$ as the input data, which first mapped to a hidden representation using the encoder:

y=φ(Wx+b) (1)

Then, it was reconstructed using a decoder,

z=φ'(W'y+b') (2)

where *W* is a weight matrix and *b* is the bias vector of the encoder, then *W*’ is a weight matrix and *b*’ is the bias vector of the decoder, and nonlinear functions were represented by *φ* and *φ*’.

To minimise the error of reconstruction, all parameters are trained to make *z* as the uncorrupted input of *x*. It can be formulated as:

$$L=\begin{array}{c} \arg\min\\ \theta \end{array}\frac{1}{N}\sum_{i=1}^{N}\|x_{i}-z_{i}\|\begin{array}{c} 2\\ 2 \end{array}$$ (3)

where *θ* is a parameter set {*W*,*b*,*W*’,*b*’}, *N* is the number of data samples, and *i* is the sample index.

The previous study proposed the ConvBiLSTM as the delineation model for the ECG single-lead [26]. The four convolution layers and BiLSTM are generated to onset and offset the P-wave, QRS-complex, T-wave, and other ECG segment classifications. In this study, we propose DAE for ECG noise cancellation and ConvBiLSTM for the delineation model. The hyperparameter tuning for DAE-ConvBiLSTM can be listed in Table 2. Table 2 listed the filter and kernel sizes, epochs, batch size, optimisation, loss function, and learning rate as the optimisation to obtain the best hyperparameter for a learning algorithm. In this study, we have tuned two DAE model with disctinct architecture. In the first model of DAE, there were 370 and 185 nodes, for encoder and decoder layers. In addition, for the second model of DAE, there were 370 nodes for the encoder and decoder layers for the DAE model, respectively. Table 2 also shows the comparison of SNR result of both the DAE model. As we can seen, the SNR of the second model of DAE achieve the highest of 36.94 decibels (dB) and the first model of DAE only get 34.28 dB. As a result, the second model outperformed of the first model of DAE. In the second model of DAE, the rectified linear unit (ReLU) is commonly used with the activation function (nonlinear) in the encoder layer and the output layer (the last layer) contains a sigmoid in the range [0, 1]. The ConvBiLSTM consisted of four convolutional layers (8, 16, 32, and 64 filter sizes), the stride of one, kernel size of three, and single layer BiLSTM. The output of DAE-ConvBiLSTM predicts the start and end of the P-wave, QRS-complex, T-wave, and isoelectric line. The pseudocode of DAE-ConvBiLSTM can be presented in algorithm 1.

**Table 2:** The hyperparameter optimisation of DAE-ConvBiLSTM.

Hyperparameters	DAE (Noise Cancellation)			ConvBiLSTM (Delineation)
	Model 1	Model 2		
Layer	(370–185) (ReLU-Sigmoid)	(370–370) (ReLU-Sigmoid)		Convolution 8 x 3, 16 x 3, 32 x 3, 64 x 3, strides = 1 + ReLU—BiLSTM
Epochs	400			300
Batch size	64			8
Optimisation	Adaptive moment estimation (Adam)			
Loss function	Mean Square Error (MSE)			Categorical cross-entropy
Learning rate	0.00095			10^−5^
Reconstructed SNR (dB)	34.28		36.94	

Algorithm 1. DAE-ConvBiLSTM

Parameter: input x(370,1), output *y*_*t*_(370,5)

1: **For** each epoch **do:**

2:    **If** a length < 370 do:

3:        Apply zero-pad to $a_{ij}^{m}$

4:        #Dimension of a is (370, Filtersize)

5:    **End if**

#Denoising Autoencoder (DAE)

6:    **For** each sample in X **do:**

7:        Calculate Encoder of a by y = *φ*(*W*_*x*_+*b*)

8:        Calculate Decoder of a by z = *φ*′(*W*′_*y*_+*b*′)

9:    **End for**

10: **End for**

11: **For** each epoch **do:**

#CNNs Feature Extraction

12:    **For** each convolutional layer **do**:

13:        **For** each sample in X **do**:

14:            Calculate $a_{ij}^{m}$ from X by

            $a_{ij}^{m}=\varphi (b_{i}+\sum_{k=1}^{M}W_{ik}\;X_{kj}+k-1)=\varphi (b_{i}+W_{i}^{T}\;X_{j})$

15:        **End for**

16:    #Dimension of a is (370—KernelSize + 1, FilterSize)

17:    **End for**

18:    #Dimension of a is (370, 64)

#BiLSTM Classifier

19:    **For** each sample in a **do:**

20:        Calculate Forward Pass of a by LST ${\vec{M}}_{ft}^{1}=tanh(W_{i\vec{h}}^{1}x_{t}+W_{\vec{h}\vec{h}}^{1}LSTM_{t-1}^{\vec{1}}+b_{\vec{h}}^{1})$

21:        Calculate Backward Pass of a by LST ${\overset{↼}{M}}_{bt}^{1}=tanh(W_{i\overset{↼}{h}}^{1}x_{t}+W_{\overset{↼}{h}\overset{↼}{h}}^{1}LSTM_{t+1}^{\overset{↼}{1}}+b_{\overset{↼}{h}}^{1})$

22:    #Dimension of the output a is (370,2*Neuron Size)

23:        Calculate y, by $y_{t}^{1}=tanh(W_{h\vec{o}}^{1}LST{\vec{M}}_{t}^{1}+W_{h\overset{↼}{0}}^{1}LST{\overset{↼}{M}}_{t}^{1}+b_{0}^{1})$

24:    **End for**

25: **End for**

To generate the DAE-ConvBiLSTM model, we have run all experiments with Windows 10 Pro 64 Bit, one Intel(R) Core (TM) i9-9900K CPU@ 3.60Ghz processor (32 RAM), and one NVIDIA GeForce RTX 2080 8GB GPU. The programming language is Python in the Spyder 5.2.2 DL framework and libraries, i.e., *tensorflow*, *keras*, *numpy*, *pandas*, *sklearn*, *matplotlib*, and the native Python WFDB package.

## Results and discussion

**Fig 2:**
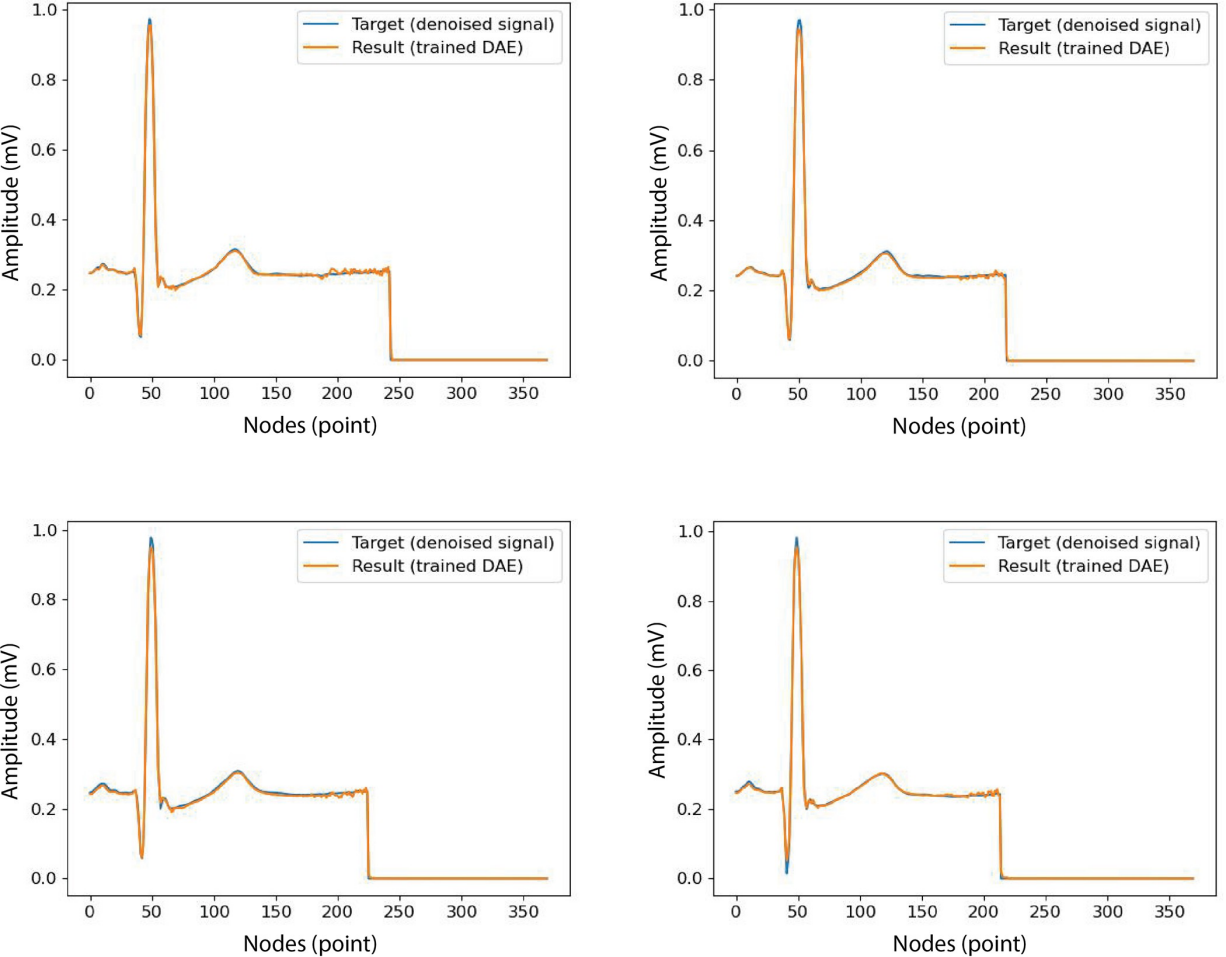
The samples of visualisation of the target and results of DAE.

The DAE algorithm was trained with noise injected with a SNR of 35 dB. The injected noise was a signal target. There were 370 input nodes representing one beat (from the start of P-wave1 to the start of P-wave2). The mean squared error (MSE) and adaptive moment estimation (Adam), with a batch size of 64, were used as a loss function and optimiser for the DAE model’s compilation (refer to Table 2). The noisy ECG sample $\tilde{x}$ is supplied to the encoder phase, which then concatenates the encoder output with the latent vector *z* and feeds it to the decoder phase. The decoder outputs the denoised ECG samples $\tilde{x}$. The trained DAE was constructed by encode and decode layers, with 370 nodes, respectively. The proposed DAE achieved 99.71% accuracy. The visualisation of the ECG signal target (denoised signal) and result (the trained DAE) can be seen in Fig 2.

The result of the trained DAE (decode layer as reconstructed signal) is used as an input of ConvBiLSTM. The delineation of the ECG signal can be classified as the start and end of the P-wave, QRS-complex, T-wave, and isoelectric line. The zero-padding is represented by class if the beat length is smaller than 370 nodes, which the technique was done by adding the value zero (0). Table 3 lists the average performance of all waveforms obtained above 98% in all metrics. Among the three main ECG waveforms, the highest accuracy and precision were achieved by the QRS-complex, corresponding to sudden depolarisation of ventricles at a rate of 99.86% and 98.57%, respectively. Our DAE-ConvBiLSTM showed promising results in detecting the R-peak. The R-peak is one of the essential sections of the QRS-complex and is used to diagnose heart rhythm abnormalities and determine heart rate variability (HRV).

**Table 3:** The performance of ECG delineation using the proposed DAE-ConvBiLSTM model.

Metrics	Performance (%)					
	P-wave	QRS-complex	T-wave	Isoelectric line	Zero-padding	Average
Accuracy	99.81	99.86	99.35	99.03	99.90	99.59
Sensitivity	98.55	99.26	97.49	98.62	100	98.79
Specificity	99.89	99.90	99.62	99.28	99.90	99.72
Precision	98.26	98.57	97.37	98.82	99.90	98.59
*F1*	98.41	98.91	97.43	98.72	99.90	98.68

**Fig 3:**
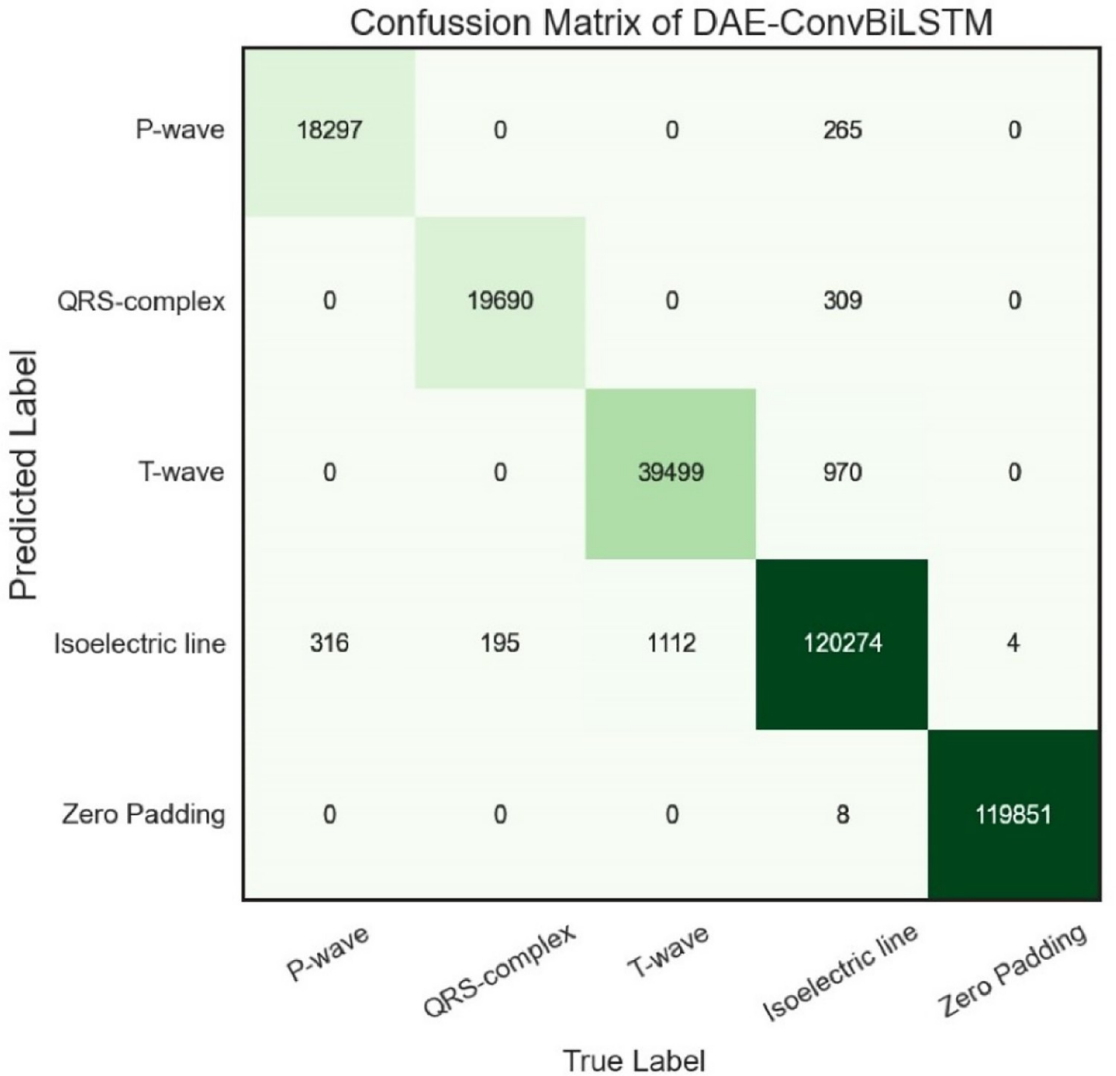
The confusion matrix of DAE-ConvBiLSTM.

To analyse the misclassified performance, the confusion matrix (error matrix) can be used to visualise the performance of a classification algorithm. Fig 3 presents the number of true and predicted labels of the P-wave, QRS-complex, T-wave, and isoelectric lines. All of the diagonal elements represent outcomes that have been correctly classified. The confusion matrix’s off diagonals represent the misclassified outcomes. As a result, the best classifier will have a confusion matrix with only diagonal members and zero values (0) for the rest of the elements. Fig 3 represents the misclassified outcomes, mostly from the isoelectric line, due to the overlapping of the ECG interval and segment around the P-wave, QRS-complex, and T-wave. The isoelectric line, or baseline (no deflections), is a straight line where there are no positive or negative charges of electricity.

**Fig 4:**
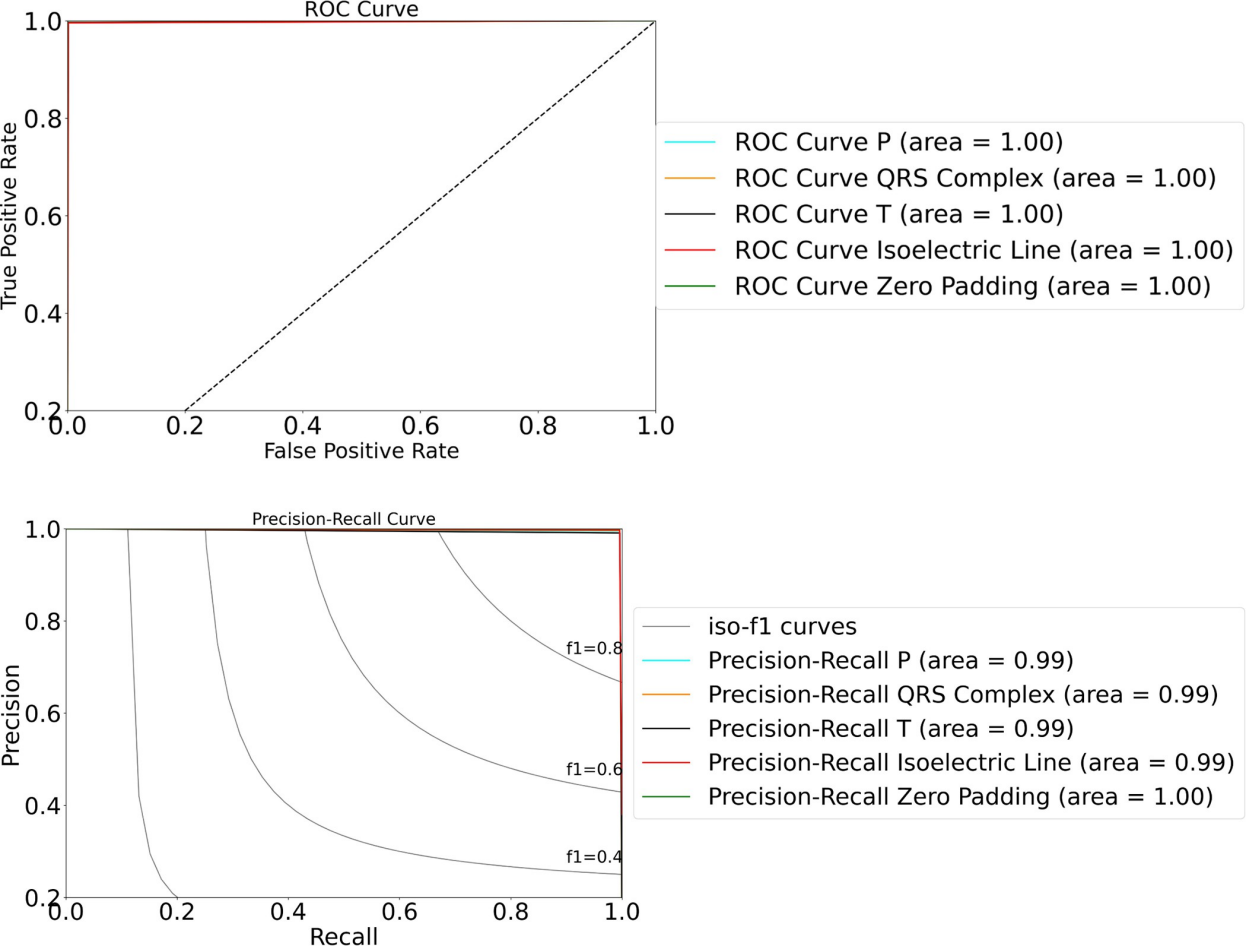
The ROC and P-R curves of DAE-ConvBiLSTM. (a) ROC curve and (b) PR curve.

A technique to visualize classifier based on their performance and to evaluate the prediction accuracy of a model, the receiver operating characteristic (ROC) curve is the most popular tool in medical research. The popularity comes from several well-studied characteristics, such as easy comparison of multiple models and the area under the curve (AUC) as a single-value quantity [32]. The application of ROC curve analysis to visualizing and examining the behavior of diagnostic systems has been extended. Fig 4(A) shows the ROC curve to visualize the DAE-ConvBiLSTM based on class of PQRST wave and isoelectric line. The AUC value has the range between 0 and 1.0, due to AUC is a portion of the area of the unit square. All class of ECG waveform present the well-performance due to the curve approaches the point 0.1.

In addition, to organize the decision problems in machine learning while dealing with highly skewed datasets, Precision-Recall (PR) curve could give a more information graph for the performance of algorithm [32]. The area under the precision-recall curve, or AUC-PR, is determined, with each point on the curve defined by a distinct value of the threshold to convert continuous to binary predictions. Different to ROC, the PR curvers plotprecision vs. recall, due to precision is influenced by imbalanced class. In imbalanced scenario, the AUC-PR will be more sensitive than AUC-ROC. Fig 4(B) shows the PR curve of all waveform class near to 1.0 (area = 0.99), as a perfect classifier. From the results of AUC-ROC and AUC-PR, it reflects the true quality of the DAE-ConvBiLSTM’s performance.

In this study, we have compared the accuracy and precision of our proposed model DAE-ConvBiLSTM to recent works related the ECG delineation using DL with QT Database (refer to Table 4). Table 4 shows that CNN and LSTM/BiLSTM have mostly explored ECG delineation tasks [33–36]. It can be claimed that CNN and LSTM/BiLSTM perform excellently in classifying three primary ECG waveforms, i.e., P-wave, QRS-complex and T-wave. However, our proposed model (DAE-ConvBiLSTM) outperformed the accuracy of P-wave, QRS-complex and T-wave in the previous studies [33, 34].

**Table 4:** The benchmarking study of recent works using DL with QT database.

Authors	Method	Performance (%)					
		Accuracy			Precision		
		P-wave	QRS-complex	T-wave	P-wave	QRS-complex	T-wave
Londhe and Atulkar [33]	CNN-BiLSTM	98.20	98.56	96.17	-	-	-
Malali *et al*. [34]	LSTM	94.87	96.66	92.73	-	-	-
Jimenez-Perex *et al*. [35]	CNN	-	-	-	90.12	99.14	98.25
Peimankar and Puthusserypady [36]	CNN-LSTM	-	-	-	-	99.52	-
Proposed work.	DAE-ConvBiLSTM	99.81	99.86	99.35	98.26	98.57	97.37

Most techniques decompose the ECG signal into a beat-to-beat time series that includes the T-wave’s characteristics. The measurement of the T-wave of the ECG signal is hard to obtain due to a precise mathematical formulation at the end of the T-wave that does not exist. However, in this study, we experiment with the ECG delineation to detect the onset and offset of the T-wave. In the case study, we detected the TWA. The TWA reflected the ECG T-wave beat-to-beat fluctuations and correlated with repolarisation dispersion and sudden cardiac arrest (SCA) mechanisms. The TWA quantification is in the range of microvolts. To detect the presence of TWA, the best model of the DAE-ConvBiLSTM is required to obtain the R-peak and T-wave. The TWA magnitude is acquired from the maximum variation between a row’s even and odd beats. After successfully separating the T-waves, the even and odd T-peaks are separated into two groups. The difference between these matrices determines whether or not it would be termed a TWA (refer to Algorithm 2). If the zero-crossing value is smaller than 0.35 times the length of the total difference between the even and odd T-peaks, the heart rate value is greater than 80 beats per minute (BPM). If the difference values tend to gather closely around some particular value with a limited number of zero-crossings, then TWA is assumed to be present.

Algorithm 2. TWA Quantification

1: initiate all_twave **as array**

2: **for** data all_t_peak **do:**

3: initiate odd_data and even_data **as float number**

4:    **If** data %2 **do**:

5:        move data to even_data

6:    **else**

7:        move data to odd_data

8:    **end if**

9:    TW = odd_data—even_data

10:    **if** zero-crossing < 0.35 * length(TW) & Heart Rate > 80

11:        all_twave (index) = max(|TW(2:end-1)|)

        #TWA detected

12:    **else**

13:        all_twave(index) = 0

        #TWA not detected

14:    **end if**

15: **end for**

We have calculated the TWA quantification using the experimented data presented in the T-Wave Alternans Challenge Database. Out of 30 records with synthesized ECGs with TWA, the DAE-ConvBiLSTM successfully achieved 20 records. The results of the TWA quantification can be listed in Table 5, which shows the calculation of zero-crossing, the difference between even and odd T-peaks, heart rate, and the results of TWA quantification. There are ten records that cannot be detected as records with the TWA’s synthesized ECGs due to overlapping between the isoelectric line. Our model has misclassified the isoelectric line to P-wave and QRS-complex, but mostly misclassified occur in T-wave (refer to Fig 3). The TWA quantification relies on precise and accurate T-wave detection. Our delineation model (ConvBiLSTM) defines the T-wave position based on *ecgpuwave* software (as the ground truth). The morphology of the T-wave tends to present an abnormal pattern, such as biphasic, inverted, and only downwards.

**Table 5:** The results of T-wave abnormalities in TWA quantification.

Records (synthesised with TWA)	Zero-crossing	Difference between even and odd T-peaks	Heart Rate	TWA Quantification	Result
twa01	31	(103,)	103	375.62	Detected
twa06	35	(123,)	123	150.68	Detected
twa09	17	(131,)	131	152.21	Detected
twa13	0	(130,)	130	18.99	Detected
twa15	48	(110,)	110	0	Non-detected
twa17	45	(124,)	124	0	Non-detected
twa21	29	(126,)	126	147.5	Detected
twa25	4	(122,)	122	2.76	Detected
twa28	50	(107,)	107	0	Non-detected
twa29	41	(131,)	131	373.89	Detected
twa30	45	(132,)	132	148.84	Detected
twa33	59	(126,)	126	0	Non-detected
twa34	18	(127,)	127	2.061	Detected
twa35	41	(133,)	133	1.726	Detected
twa50	34	(127,)	127	1.375	Detected
twa51	39	(130,)	130	1.374	Detected
twa64	29	(126,)	126	1.374	Detected
twa67	37	(128,)	128	147.764	Detected
twa69	38	(103,)	103	0	Non-detected
twa70	24	(123,)	123	148.095	Detected
twa72	0	(133,)	133	6.226	Detected
twa73	1	(124,)	124	5.186	Detected
twa76	34	(125,)	125	1.374	Detected
twa78	54	(126,)	126	0	Non-detected
twa79	33	(134,)	134	3.436	Detected
twa82	3	(130,)	130	3.453	Detected
twa88	42	(106,)	106	0	Non-detected
twa91	43	(110,)	110	0	Non-detected
twa97	60	(126,)	126	0	Non-detected
twa98	59	(126,)	126	0	Non-detected

## Conclusion

Interference or noise (unwanted signals) can contaminate an ECG with external and internal physiological processes in the body, and the morphology changes over time. The presence of unwanted signals arduous extracting accurate features from the ECG signal. It would affect the reliability of diagnosing heart abnormalities in clinical practices. This paper aims to develop an end-to-end learning algorithm for heart abnormality detection, using unsupervised learning DAE and supervised learning ConvBiLSTM. The proposed method, DAE-ConvBiLSTM, has been implemented to detect the abnormality of T-waves related to heart abnormalities (due to ventricular repolarisation). As a result, for the ECG denoising algorithm, DAE has obtained 99.71% accuracy. The DAE reconstruction is learned, in which the AE attempts to produce a representation as close to its original input as possible from the reduced encoding. It may aid the AE’s understanding of the main data features. The AE algorithm is progressively being used to learn generative models of data. In addition, the DAE-ConvBiLSTM has successfully achieved an average performance above 98% in all performance metrics. The proposed DAE-ConvBiLSTM can therefore detect the T-wave abnormality related to TWA.

Although the results of our study look promising, some limitations can be explored in the future:

1. To generate the DAE-ConvBiLTM, we only used the single ECG database with the normal sinus rhythm records with the complete and normal pattern of the ECG waveform.
2. The proposed model, DAE-ConvBiLSTM, has only been tested to detect the presence of TWA. In the future work, there will be more chances to enhance the performance of end-to-end learning to detect several heart abnormalities related to ECG morphology besides TWA. The precise accuracy of the ECG’s main waveform will affect the detection of heart abnormalities in clinical practice.
